# *Opuntia humifusa* Supplementation Increased Bone Density by Regulating Parathyroid Hormone and Osteocalcin in Male Growing Rats

**DOI:** 10.3390/ijms13066747

**Published:** 2012-06-04

**Authors:** Junyong Kang, Jinho Park, Seong Hee Choi, Shoji Igawa, Youngju Song

**Affiliations:** 1 Laboratory of Sports Nutrition, Sunmoon University, Asan, 336-708, Republic of Korea; E-Mails: garcia60@sunmoon.ac.kr (J.K.); jinho0418@hanmail.net (J.P.); 2 Department of Food Science, Sunmoon University, Asan, 336-708, Republic of Korea; E-Mail: choish@sunmoon.ac.kr; 3 Department of Healthcare, Nippon Sport Science University, Yokohama, 227-0033, Japan; E-Mail: igawa@nittai.ac.jp

**Keywords:** *O. humifusa*, bone density, parathyroid hormone, osteocalcin, rat

## Abstract

We investigated the effect of *Opuntia humifusa* (*O. humifusa*) supplementation on bone density and related hormone secretion in growing male rats. Sixteen six-week-old male Sprague-Dawley rats were randomly divided into two groups; control diet group (CG, *n* = 8), and experimental diet group (EG, *n* = 8). The rats in the CG were given a control diet and those in the EG were given 5% *O. humifusa* added to the control diet for eight weeks. The serum OC level of the EG was significantly higher than that of the CG, and the serum parathyroid hormone (PTH) level of EG was significantly lower than that of the CG. In addition, the femoral and tibial BMD of the EG were significantly higher values than those of the CG, and the tibial BMC of the EG was significantly higher than that of the CG. These results suggest that *O. humifusa* supplementation has a positive effect on bone density by suppressing PTH and increasing the OC level in growing male rats.

## 1. Introduction

Osteoporosis is one of the most common metabolic bone diseases, and it increases the risk of fracture by reducing bone strength [1]. Although the period during which peak bone mass is attained differs according to bone type, most bones grow rapidly through puberty, and peak bone mass is attained at approximately 25 years old, after which bone mass is gradually decreased [2]. However, previous studies have suggested that the bone mass is not only relatively decreased but also at a higher risk of fracture in obese children because the bone mineral density (BMD) and bone mineral content (BMC) are lower in obese children than in normal children [3,4]. Nevertheless, sufficient mineral intake in the growth period, such as calcium (Ca^2+^) [5] and magnesium (Mg^2+^) [6], can reduce the risk of fracture by increasing BMD and BMC, and can also reduce the risk of osteoporosis or fracture in elderly individuals [7].

Among the mineral nutrients, Mg^2+^ is essential for physiological function in various tissues and organs [8]. In particular, Mg^2+^ deficiency is well known as a potential risk factor of osteoporosis [9], as well as a cause of impaired parathyroid hormone (PTH) secretion, which in turn dysregulates Ca^2+^ homeostasis [10]. Furthermore, because Mg^2+^ depletion has a negative effect on the synthesis of 1,25(OH)_2_ vitamin-D, bone health is negatively influenced by low Mg^2+^ intake [11]. In addition, in the relationship between Ca^2+^ and bone metabolism, PTH secretion is increased due to Ca^2+^ deficiency-induced hypocalcemia, and serum Ca^2+^ level is rapidly restored by PTH stimulating osteoclasts in the bone [12]. Therefore, fracture risk factors, such as increased bone turnover and bone loss, are influenced by excessive PTH secretion [13,14].

Meanwhile, *Opuntia humifua* (*O. humifusa*), which is a perennial plant, is a member of the Cactaceae family that grows mostly in semi-arid regions all around the world, particularly in Central and South America. In particular, *O. humifusa* has been cultivated in large quantities in Asan, Chungnam, Korea. The few studies that have investigated the physiological and pharmacological functions of *O. humifusa* have revealed not only its radical scavenging and anti-inflammatory effects [15] but also hypoglycemic and hypolipidemic effects [16]. On the other hand, it has been reported that *Opuntia ficus-indica (O. ficus-indica)*, another member of the Cactaceae family, has anti-inflammatory [17], anti-cancer [18], and antioxidant effects [19], and many studies of its nutritional and pharmacological aspects have demonstrated that it contains high levels of Mg^2+^ and Ca^2+^ [20]. However, although we may anticipate that *O. humifusa* also contains various minerals, few studies investigated this aspect of *O. humifusa* compared with *O. ficus-indica*. In particular, there have been no studies designed to examine the relationship between *O. humifusa* and bone metabolism.

Therefore, in this study, we investigated the effects of *O. humifusa* supplementation on bone-related hormones and bone metabolism.

## 2. Results

### 2.1. Body Weight, FER and Food Intake

As shown in Table 1, although the average body weight was the same in both groups at the beginning of the study, final body weight was slightly decreased in the EG compared with the CG after eight weeks of *O. humifusa* supplementation. Additionally, the FER values and food intake were not significantly different between groups.

### 2.2. Changes in Serum Parameters

After the eight weeks of the experiment, as shown in Table 2, there were no significant differences in the Ca, P and ALP values between groups. However, of the hormone concentrations related to bone metabolism, the OC level was significantly higher in the EG than in the CG (*p <* 0.05), and the PTH value was significantly lower in the EG than in the CG (*p <* 0.05).

### 2.3. Bone Weight, BMD and BMC

**Figure 1:**
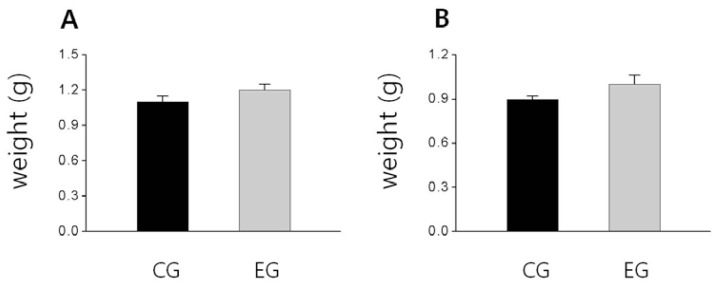
The effect of *O. humifusa* supplementation on bone weight. The data were expressed as the mean ± SE. (**A**) Femur, (**B**) Tibia. CG: Control diet group; EG: Experimental diet group.

**Figure 2:**
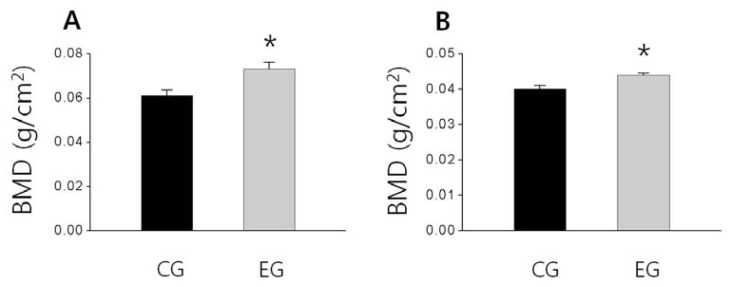
The effect of *O. humifusa* supplementation on bone mineral density. The data were expressed as the mean ± SE. (**A**) Femur, (**B**) Tibia. CG: Control diet group; EG: Experimental diet group. * *p <* 0.05 compared with the CG.

**Figure 3:**
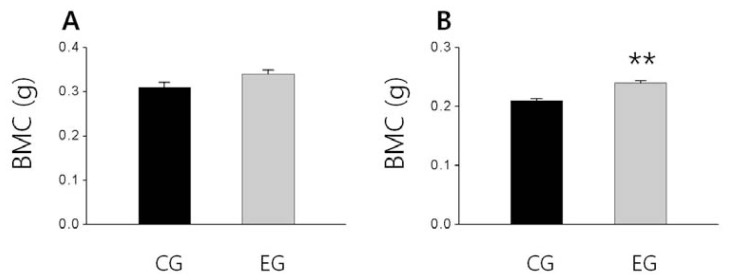
The effect of *O. humifusa* supplementation on bone mineral content. The data were expressed as the mean ± SE. (**A**) Femur, (**B**) Tibia. CG: Control diet group; EG: Experimental diet group. ** *p <* 0.01 compared with the CG.

As shown in Figure 1, the femoral bone weight in the EG tended to be higher than that of the CG and also the tibial bone weight slightly higher than that of the CG, but these difference were not significant, respectively (Figure 1A,B). Additionally, the BMD of the femur and tibia of EG were significantly higher than that of the CG (*p <* 0.05; Figure 2A, *p <* 0.05; Figure 2B). The tibial BMC of the EG was significantly higher than that of the CG (*p <* 0.01; Figure 3B), and the femoral BMC of the EG tended to be higher than that of the CG (Figure 3A).

## 3. Discussion

To the best of our knowledge, this is the first study to evaluate the effects of *O. humifusa* supplementation on bone metabolism. Our results indicated that serum Ca, P and ALP levels were not significantly affected after eight weeks of treatment. However, the OC level of the EG was significantly higher than that of the OC of the CG. OC, a bone formation marker, is a major noncollagenous protein in the bone matrix that is synthesized by and released from osteoblasts [21]. Approximately 70% of the OC released from osteoblasts is combined with bone matrix and dentin cells, and the remaining 30% of the OC is secreted into the blood [22]. In a previous study, however, even though BMD was higher in rats with high OC levels [23], insufficient intake of minerals, such as Mg^2+^, lead to a reduction of serum OC levels [24]. Furthermore, OC is also correlated with Ca^2+^ levels, and approximately 99% of Ca^2+^ in the human body, which exists as hydroxyapatite in bones, is involved in maintaining skeletal structural strength [25]. OC has a high affinity for calcium and exhibits a compact-calcium dependent α-helical conformation, in which the gamma-carboxylated glutamates binds and promote absorption as hydroxyapatite in the bone matrix, leading to bone mineralization [26]. Therefore, intake of sufficient minerals, including Mg^2+^ and Ca^2+^, promote an increase in OC level [27]. As a result, we might assume that supplementation with *O. humifusa*, which contains Mg^2+^ and Ca^2+^, would have a positive effect on bone formation and the achievement of peak bone mass in the growth period by increasing the levels of the osteogenic marker OC in the EG compared with CG.

At the same time, the PTH of the EG was significantly lower than that of the CG. Serum Ca^2+^ is well known to be regulated by PTH and vitamin D. When serum Ca^2+^ is low, increased PTH secretion stimulates calcium mobilization through bone resorption by osteoclasts [28]. Another mechanism for restoring the serum Ca^2+^ level to the normal range is by decreasing the excretion of Ca^2+^, which requires Ca^2+^ absorption in the intestine; this is promoted by an increased formation of 1,25(OH)_2_ vitamin-D by the activation of 25(OH) vitamin D-1α hydroxylase in the kidney. Therefore, because dietary calcium restrictions might cause hypocalcemia-induced osteoporosis due to increased bone resorption as well as decreased bone mass [29], repletion of calcium supplementation might have a beneficial effect on bone metabolism. In fact, Reid *et al*., reported that bone loss was delayed by 0.5~1% by the intake of over 800 mg/day calcium compared with intake below 800 mg/day for a period of 1~2 years in females with progressive bone loss [30]. In addition, a previous report has shown that 1000 mg/day calcium and 800 IU/day vitamin D supplementation significantly reduces PTH levels and increases BMD in the context of Mg^2+^ deficiency in females over the age of 65 [31]. Therefore, our data are consistent with previous findings, suggesting that *O. humifusa* provides sufficient Ca^2+^ and might have beneficial effects on the maintenance of the structural strength of bone, suppressing the calcium mobilization from bone by arresting PTH secretion.

Furthermore, it has been reported that Mg^2+^ plays a crucial role in vitamin D metabolism [32]. Serum 1,25(OH)_2_ vitamin-D concentrations are frequently low in patients with Mg^2+^ deficiency because Mg^2+^ deficiency is thought to have a negative effect on vitamin D metabolism [32]. Thus, PTH and vitamin D are essential for the physiological function of osteoblasts [33], and changes such as an increase in PTH and a reduction of 1,25(OH)_2_ vitamin-D may impair bone formation. In fact, some studies reported that diet-induced Mg^2+^ deficiency causes not only reduced bone mass but also skeletal fragility in a rodent model [34,35]; the observed reduction of bone mass may have been due to a reduction in the number of osteoblasts [36] by Mg^2+^ deficiency. However, in a previous study, 0.15% Mg^2+^ supplementation suppressed bone resorption due to declining PTH levels in ovariectomized rats [27]. Similarly, Stending-Lindberg *et al*., reported that postmenopausal women receiving two to six 125 mg tablets of magnesium hydroxide daily for six months and two tablets for 18 months in a two year period exhibited increased trabecular bone density or arrested bone loss [9].

Therefore, as mentioned above, a reduction of bone formation and increase of bone resorption might be caused by the impairment of mineral intake as well as by Mg^2+^ and Ca^2+^ deficiency, and could play a negative role in bone growth. However, sufficient intake of individual nutrients such as Ca^2+^ and Mg^2+^, or functional drinks that contain replenishing minerals have a beneficial effect on BMD and BMC [37,38]. According to Kang *et al*., in a study of the changes of bone metabolism in growing rats caused by Ca^2+^ and Mg^2+^, long-term supplementation with deep-sea water, which is rich in Mg^2+^ and Ca^2+^, improves bone metabolism, as reflected by increased BMD, BMC and the breaking force of bones [38]. These results supported those of our present study; we calculated that the experimental diet group received approximately 2.2 times the level of Mg^2+^ and 1.3 times the level of Ca^2+^ as the control group due to the 5% *O. humifusa* added to the experimental diet. The mineral-rich *O. humifusa* supplement likely exerted positive effects on bone metabolism through the suppression of PTH secretion as well as increased intestinal Ca^2+^ absorption due to the activation of 1,25(OH)_2_ vitamin-D in the kidney. Additionally, further studies, such as the bone resorption markers or bone histomorphological study, are also needed to more elucidate the effect of *O. humifusa* supplementation on bone metabolism.

## 4. Materials and Methods

### 4.1. Experimental Animals

All experimental protocols were approved by the Animal Study Committee of Sunmoon University. After a one-week acclimatization period, sixteen six-week-old male Sprague-Dawley rats (Samtaco Bio Korea, Hwaseong, Korea) were randomly divided into two groups, a control diet group (CG, *n* = 8) and an experimental diet group (EG, *n* = 8) and given free access to tap water for eight weeks. Rats were housed in groups of two per cage under controlled temperature (23 ± 1 °C) and relative humidity (50 ± 5%). The light/dark cycle was automatically controlled (alternating 12-h periods) and the dark period began at 8:00 am. Food intake was measured daily and body weight was measured weekly. Food efficiency ratio (FER) was calculated as the total weight gained, divided by the total food intake for the experimental period. At the end of the experimental period, the rats were anesthetized with diethyl ether after fasting for 12 h. Blood samples were taken from the left ventricle and serum was obtained by centrifuging the blood at 700× *g* for 20 min at 4 °C. Serum samples were stored at −70 °C until further analysis.

### 4.2. Preparation of Experimental Diet

*O. humifusa* was harvested in Asan, Chungnam, washed and blended using a HMF-3150S blender (Hanil Electronics, Seoul, Korea). After blending, the *O. humifusa* was frozen at −70 °C and then freeze-dried (Ilshin Co., Gyeonggi, Korea). After freeze-drying, component analysis of *O. humifusa* was performed by Eco-Bio Korea (Gyeonggi, Korea); the results of this analysis are shown in Table 3. The composition of the control diet, which was based on AIN-76G, was 20% protein, 48% carbohydrate and 20% fat, as modified in a previous study [39]. The 5% *O. humifusa* diet was made by substituting a portion of the carbohydrate, protein, fiber, and fat components of the control diet. During the experimental period, the diets was prepared fresh in 3~5 day batches, and stored at 4 °C to maintain freshness.

### 4.3. Serum Analysis

Serum Ca, P, and alkaline phosphatase (ALP) concentrations were measured using a TBA-200FR automated analyzer (Toshiba, Tokyo, Japan), and serum osteocalcin. (OC) was analyzed by radioimmunoassay (RIA) method using the osteocalcin MYRIA (TECHNO GENETICS, Milano, Italy) assay kit. Serum concentrations of PTH were determined using the rat parathormone i-PTH ELISA kit (Wuhan EIAab Science Co., Ltd., Wuhan, China).

### 4.4. Bone Weight, BMD and BMC Analysis

Both the femur and tibia were obtained from the lower limbs, and the samples were stored in 70% ethyl alcohol until bone weight, BMD and BMC measurements were made. The bone weight was measured using an AR-2140 analytical balance (OHAUS Corporation, NJ, USA), and the BMD and BMC of the femur and tibia were measured with dual-energy X-ray absorptiometry (DEXA, Lunar DPXL, software version 1.0C; Madison, WI, USA).

### 4.5. Statistical Analysis

All data were analyzed using SPSS software (version 15.0 for Windows). The data are expressed as the mean ± SE, and values were analyzed with the independent samples *t* test. Significance was defined as *p <* 0.05.

## 5. Conclusions

The results of the present study suggest that *O. humifusa* can be considered a functional food for the improvement of bone strength due to its ability to increase bone density via the suppression of PTH and increase of OC level.

## Figures and Tables

**Table 1:** Changes in body weight, FER and food intake.

	CG	EG
Initial body weight (g)	235.9 ± 3.89	235.8 ± 3.97
Final body weight (g)	428.2 ± 10.86	419.3 ± 14.67
FER	0.26 ± 0.008	0.26 ± 0.011
Food intake (g/day)	16.2 ± 0.38	14.9 ± 0.16

Data were expressed as the mean ± SE; CG: Control diet group; EG: Experimental diet group; FER: Food efficiency ratio; ** *p <* 0.01 compared with the CG.

**Table 2:** Changes in serum parameters.

	CG	EG
Ca (mg/dL)	10.5 ± 0.14	10.2 ± 0.21
P (mg/dL)	7.4 ± 0.33	7.6 ± 0.17
ALP (IU/L)	87.0 ± 2.55	91.0 ± 3.18
OC (ng/mL)	25.8 ± 0.53	30.4 ± 1.41 *
PTH (pg/mL)	13.7 ± 0.88	10.7 ± 0.45 *

Data were expressed as the mean ± SE; CG: Control diet group; EG: Experimental diet group; Ca: Calcium; P: Phosphorus; ALP: Alkaline phosphatase; OC: Osteocalcin; PTH: Parathyroid hormone;

* *p <* 0.05 compared with the CG.

**Table 3:** Components of *O. humifusa*.

Ingredients	Contents
moisture (% *w*/*w*)	2.89
ash (% *w*/*w*)	13.8
carbohydrate (g/100 g)	46.56
crude protein (g/100 g)	4.91
crude fat (g/100 g)	3.06
fiber (g/100 g)	28.78
Fe^2+^ (mg/g)	5.76
Ca^2+^ (mg/100 g)	2931.3
Mg^2+^ (mg/100 g)	1227.9
K^+^ (mg/100 g)	2155.5
Na^+^ (mg/100 g)	30.9
P^2+^ (mg/100 g)	653.2
